# Crystal structures of titanium–aluminium and –gallium complexes bearing two *μ_2_*-CH_3_ units

**DOI:** 10.1107/S2056989017004856

**Published:** 2017-04-13

**Authors:** Tim Oswald, Mira Diekmann, Annika Frey, Marc Schmidtmann, Rüdiger Beckhaus

**Affiliations:** a Institut für Chemie, Fakultät für Mathematik und Naturwissenschaften, Carl von Ossietzky Universität Oldenburg, 26129 Oldenburg, Germany

**Keywords:** crystal structure, titanium metallocene, titanocene, tri­methyl­aluminium, tri­methyl­gallium

## Abstract

The isotypic crystal structures of two titanocene complexes containing an *E*Me_3_ unit (*E =* Al, Ga; Me = meth­yl) with two *μ*_2_-coordinating methyl groups, namely [μ-1(η^5^)-(adamantan-1-yl-2κ*C* ^1^)cycylo­penta­dien­yl]di-μ_2_-methyl-methyl-2κ*C*-[1(η^5^)-penta­methyl­cyclo­penta­dien­yl]aluminiumtitanium(III), [AlTi(CH_3_)_3_(C_10_H_15_)(C_15_H_18_)], and [μ-1(η^5^)-(adamantan-1-yl-2κ*C* ^1^)cycylo­penta­dien­yl]di-μ_2_-methyl-methyl-2κ*C*-[1(η^5^)-penta­methyl­cyclo­penta­dien­yl]galliumtitanium(III), [GaTi(CH_3_)_3_(C_10_H_15_)(C_15_H_18_)], are reported. Reacting a dinuclear nitro­gen-bridged low-valent titanium(III) complex with the Lewis acids AlMe_3_ or GaMe_3_ results in the loss of mol­ecular di­nitro­gen and the formation of two monomeric titanocene(III) fragments bearing two *μ*_2_-bridging methyl groups. Single crystal X-ray diffraction reveals the formation of a new *E*—C bond involving the penta­fulvene ligand while the bridging and terminal methyl groups remain intact.

## Abstract 2

The mol­ecular structures of two isotypic titanium(III) complexes bearing an tri­methyl­aluminium or -gallium motif are reported. In both compounds, two methyl groups coordinate to the metal atoms, *viz*. Ti and Al(Ga), and in a *μ*_2_ manner.

## Chemical context   

Tri­methyl­aluminium, AlMe_3_, is of great inter­est because of its use in the synthesis of methyl­aluminoxane as co-catalyst in olefin polymerization (Wang, 2006 ▸; Janiak, 2006 ▸). In organometallic chemistry, many reactions involving tri­methyl­aluminium have been investigated, *e.g.* the Tebbe reagent Cp_2_ZrCl(CH_2_Al(CH_3_)_2_) (Cp = cyclo­penta­dien­yl), which can be used for methyl­ation reactions (Tebbe *et al.*, 1978 ▸; Thompson *et al.*, 2014 ▸). Employing multiple C—H activation reactions, the formation of zirconium- or hafnium-containing clusters [(Cp*M)_3_Al_6_Me_8_(CH_2_)_2_(CH)_5_] (*M* = Zr, Hf) have been described (Herzog *et al.*, 1996 ▸). In a similar manner, the formation of [TiAl(C)CH_3_] or [TiAl(CH_2_)_2_] metallacycles have been reported (Kickham *et al.*, 2002 ▸; Stephan, 2005 ▸). It is noteworthy that all these complexes result from C—H activation reactions. Since bond activation reactions employing penta­fulvene-substituted metal complexes have been of great inter­est in our work group (Oswald *et al.*, 2016 ▸; Manssen *et al.*, 2015 ▸; Ebert *et al.*, 2014 ▸), we therefore investigated the reactivity of a dinuclear nitro­gen-bridged penta­fulvene titanium complex towards AlMe_3_ and its heavier analogue GaMe_3_. Here we report on syntheses and crystal structures of the resulting title compounds, **1** and **2**.

## Structural commentary   

**Figure 1:**
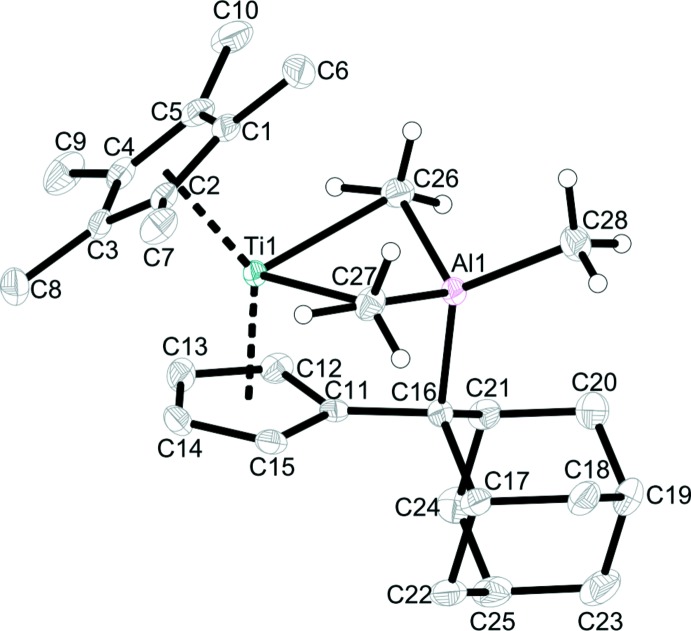
The mol­ecular structure of complex **1**. Displacement ellipsoids correspond to the 50% probability level. H atoms have been omitted for clarity except for those of methyl groups C26, C27 and C28.

Figs. 1 ▸ and 2 ▸ show the mol­ecular structures of **1** and isotypic **2**, respectively. Both complexes show the formation of a titanium tri­methyl­aluminium or -gallium metallacycle, in which the *E*Me_3_ units are still intact and exhibit a *μ*_2_-bridging mode of the methyl groups. Additionally, a new C—Al/Ga bond is formed and the former double bond C11—C16 of the penta­fulvene ligand is repealed and at 1.509 (2) Å (**1**), or 1.507 (2) Å (**2**) is within the range of a single bond (1.53 Å; March, 2007 ▸). As a result of this coordination, the tetra­valent aluminium and gallium atoms differ from the ideal tetra­hedral conformation.
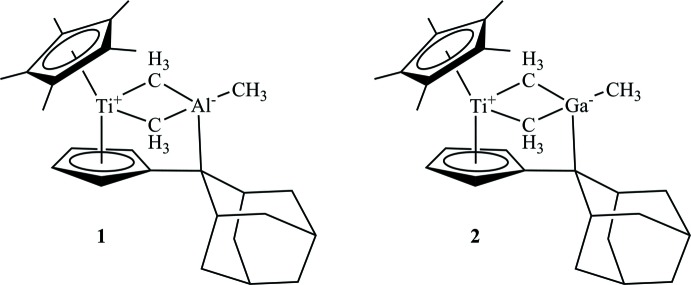


The bond lengths Al1—C26 [2.028 (2) Å] and Al1—C27 [2.047 (2) Å] in **1** are significantly elongated in comparison with that to the terminal methyl group [1.969 (2) Å], but are in good agreement with those of the free Al_2_Me_6_ mol­ecule (Vranka & Amma, 1967 ▸). The same behaviour can be observed in **2** where the Ga1—C26 and Ga1—C27 distances [2.056 (2) and 2.099 (2) Å, respectively] are elongated compared to the Ga1—C28 bond length of 1.987 (2) Å [1.966 (2) Å in GaMe_3_; Beagley & Schmidling, 1974 ▸]. The Ti—C26 [2.546 (2) Å] and Ti—C27 [2.507 (2) Å] distances in **1** are significantly longer than terminal Ti—CH_3_ distances, *e.g*. Cp_2_TiMe_2_ (*ca* 2.16 Å; Thewalt & Wöhrle, 1994 ▸) or bridging Ti—CH_3_ distances such as in [Ti(NtBu)(Me_3_[9]aneN_3_)(*μ*-Me)_2_AlMe_2_]^+^ (*ca* 2.3 Å; Bolton *et al.*, 2005 ▸).

## Supra­molecular features   

**Figure 3:**
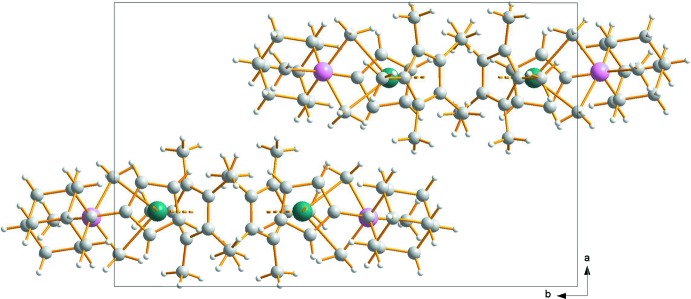
A view along the *c* axis showing the packing of mol­ecules in the crystal structure of compound **1**. No significant supra­molecular features can be observed. Colour code: C grey, H white, Al pink, Ti turquoise spheres.

For both complexes, no significant supra­molecular features are observed. The crystal packing (Fig. 3 ▸) appears to be dominated by van der Waals inter­actions.

## Synthesis and crystallization   

All reactions were carried out under a dry nitro­gen atmosphere using Schlenk techniques or in a glove box. The starting titanium complex was prepared according to a published procedure (Scherer *et al.*, 2005 ▸). AlMe_3_ and GaMe_3_ solutions were purchased from Sigma Aldrich and used as received. Solvents were dried according to standard procedures over Na/K alloy with benzo­phenone as indicator and distilled under a nitro­gen atmosphere.

**Synthesis of 1:**

Bis[(*η* ^5^-penta­methyl­cyclo­penta­dien­yl)(*η* ^5^:*η* ^1^-adamantylidene­penta­fulvene)titanium]-*μ* ^2^,*η* ^1^,*η* ^1^-di­nitro­gen (500 mg, 0.632 mmol) was dissolved in toluene and AlMe_3_ (2 *M* solution in toluene, 0.65 ml, 1.3 mmol) was added. The colour of the solution changed from blue to green, after 48 h the volume had reduced to 5 ml and another 5 ml of *n*-hexane were added. Crystals suitable for X-ray diffraction separated after 48 h directly from the mother liquor.

**Synthesis of 2:**

Bis[(*η* ^5^-penta­methyl­cyclo­penta­dien­yl)(*η* ^5^:*η* ^1^-adamantylidene­penta­fulvene)titanium]-*μ* ^2^,*η* ^1^,*η* ^1^-di­nitro­gen (100 mg, 0.13 mmol) was dissolved in toluene and GaMe_3_ (1.7 *M* solution in toluene, 0.15 ml, 0.25 mmol) was added. The former blue solution turned brown and was stored at 233 K. After 10 days, brown–green crystals suitable for X-ray diffraction separated from the mother liquor.

## Refinement   

Crystal data, data collection and structure refinement details are summarized in Table 1 ▸. Hydrogen atoms bonded to C atoms were located from difference-Fourier maps but were subsequently fixed to idealized positions using appropriate riding models with *U*_iso_(H) = 1.2*U*_eq_(C); H atoms of all methyl groups were refined freely.

## Supplementary Material

Crystal structure: contains datablock(s) global, 1, 2. DOI: 10.1107/S2056989017004856/wm5378sup1.cif

Structure factors: contains datablock(s) 1. DOI: 10.1107/S2056989017004856/wm53781sup2.hkl

Structure factors: contains datablock(s) 2. DOI: 10.1107/S2056989017004856/wm53782sup3.hkl

CCDC references: 1540662, 1540661

Additional supporting information:  crystallographic information; 3D view; checkCIF report

## Figures and Tables

**Figure 2:**
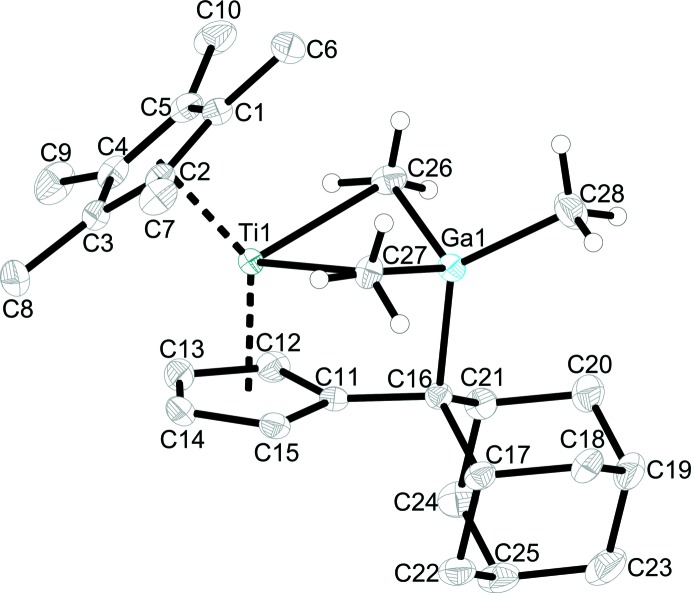
The mol­ecular structure of complex **2**. Displacement ellipsoids correspond to the 50% probability level. H atoms have been omitted for clarity except for those of methyl groups C26, C27 and C28.

**Table 1:** Experimental details

	**1**	**2**
Crystal data		
Chemical formula	[AlTi(CH_3_)_3_(C_10_H_15_)(C_15_H_18_)]	[GaTi(CH_3_)_3_(C_10_H_15_)(C_15_H_18_)]
*M*_r_	453.49	496.23
Crystal system, space group	Monoclinic, *P*2_1_/*c*	Monoclinic, *P*2_1_/*c*
Temperature (K)	153	153
*a*, *b*, *c* (Å)	12.1618 (5), 19.8355 (8), 10.0403 (6)	12.1445 (8), 19.9196 (7), 10.0350 (4)
β (°)	91.417 (6)	91.400 (7)
*V* (Å^3^)	2421.3 (2)	2426.9 (2)
*Z*	4	4
Radiation type	Mo *K*α	Mo *K*α
μ (mm^−1^)	0.40	1.45
Crystal size (mm)	0.55 × 0.18 × 0.11	0.50 × 0.30 × 0.29

Data collection		
Diffractometer	Stoe IPDS	Stoe IPDS
Absorption correction	–	Numerical (*X-RED*; Stoe, 1999 ▸)
*T*_min_, *T*_max_	–	0.571, 0.717
No. of measured, independent and observed [*I* > 2σ(*I*)] reflections	24801, 4572, 3201	28356, 5895, 4830
*R*_int_	0.068	0.042
(sin θ/λ)_max_ (Å^−1^)	0.617	0.668

Refinement		
*R*[*F* ^2^ > 2σ(*F* ^2^)], *wR*(*F* ^2^), *S*	0.031, 0.068, 0.87	0.026, 0.065, 0.94
No. of reflections	4572	5895
No. of parameters	295	295
H-atom treatment	H atoms treated by a mixture of independent and constrained refinement	H atoms treated by a mixture of independent and constrained refinement
Δρ_max_, Δρ_min_ (e Å^−3^)	0.38, −0.22	0.57, −0.32

Computer programs: *IPDS* and *X-RED* (Stoe, 1999 ▸), *SHELXS97* (Sheldrick, 2008 ▸), *SHELXL2014/7* (Sheldrick, 2015 ▸), *DIAMOND* (Brandenburg & Putz, 2006 ▸) and *publCIF* (Westrip, 2010 ▸).

## supplementary crystallographic information

### (1) [µ-1(η5)-(Adamantan-1-yl-2κC1)cycylopentadienyl]di-µ2-methyl-methyl-2κC-[1(η5)-pentamethylcyclopentadienyl]-aluminiumtitanium(III) Crystal data

**Table:**

[AlTi(CH_3_)_3_(C_10_H_15_)(C_15_H_18_)]	*F*(000) = 980
*M**_r_* = 453.49	*D*_x_ = 1.244 Mg m^−^^3^
Monoclinic, *P*2_1_/*c*	Mo *K*α radiation, λ = 0.71073 Å
*a* = 12.1618 (5) Å	Cell parameters from 7286 reflections
*b* = 19.8355 (8) Å	θ = 2.4–26.0°
*c* = 10.0403 (6) Å	µ = 0.40 mm^−^^1^
β = 91.417 (6)°	*T* = 153 K
*V* = 2421.3 (2) Å^3^	Block, yellow
*Z* = 4	0.55 × 0.18 × 0.11 mm

### (1) [µ-1(η5)-(Adamantan-1-yl-2κC1)cycylopentadienyl]di-µ2-methyl-methyl-2κC-[1(η5)-pentamethylcyclopentadienyl]-aluminiumtitanium(III) Data collection

**Table:**

Stoe IPDS diffractometer	*R*_int_ = 0.068
Radiation source: sealed tube	θ_max_ = 26.0°, θ_min_ = 2.3°
ω scans	*h* = −14→14
24801 measured reflections	*k* = −24→24
4572 independent reflections	*l* = −12→12
3201 reflections with *I* > 2σ(*I*)	

### (1) [µ-1(η5)-(Adamantan-1-yl-2κC1)cycylopentadienyl]di-µ2-methyl-methyl-2κC-[1(η5)-pentamethylcyclopentadienyl]-aluminiumtitanium(III) Refinement

**Table:**

Refinement on *F*^2^	0 restraints
Least-squares matrix: full	Hydrogen site location: mixed
*R*[*F*^2^ > 2σ(*F*^2^)] = 0.031	H atoms treated by a mixture of independent and constrained refinement
*wR*(*F*^2^) = 0.068	*w* = 1/[σ^2^(*F*_o_^2^) + (0.035*P*)^2^] where *P* = (*F*_o_^2^ + 2*F*_c_^2^)/3
*S* = 0.87	(Δ/σ)_max_ = 0.002
4572 reflections	Δρ_max_ = 0.38 e Å^−^^3^
295 parameters	Δρ_min_ = −0.22 e Å^−^^3^

### (1) [µ-1(η5)-(Adamantan-1-yl-2κC1)cycylopentadienyl]di-µ2-methyl-methyl-2κC-[1(η5)-pentamethylcyclopentadienyl]-aluminiumtitanium(III) Special details

**Table:**

Geometry. All esds (except the esd in the dihedral angle between two l.s. planes) are estimated using the full covariance matrix. The cell esds are taken into account individually in the estimation of esds in distances, angles and torsion angles; correlations between esds in cell parameters are only used when they are defined by crystal symmetry. An approximate (isotropic) treatment of cell esds is used for estimating esds involving l.s. planes.

### (1) [µ-1(η5)-(Adamantan-1-yl-2κC1)cycylopentadienyl]di-µ2-methyl-methyl-2κC-[1(η5)-pentamethylcyclopentadienyl]-aluminiumtitanium(III) Fractional atomic coordinates and isotropic or equivalent isotropic displacement parameters (Å^2^)

**Table:**

	*x*	*y*	*z*	*U*_iso_*/*U*_eq_	
Ti1	0.26484 (3)	0.59327 (2)	0.23454 (3)	0.01418 (9)	
Al1	0.24338 (5)	0.45093 (3)	0.21686 (5)	0.01685 (13)	
C1	0.26433 (17)	0.62640 (9)	0.00582 (17)	0.0194 (4)	
C2	0.35839 (16)	0.65709 (9)	0.06987 (16)	0.0174 (4)	
C3	0.31849 (17)	0.70394 (9)	0.16359 (17)	0.0190 (4)	
C4	0.20285 (17)	0.70296 (9)	0.15852 (17)	0.0208 (4)	
C5	0.16835 (17)	0.65619 (10)	0.05950 (18)	0.0225 (4)	
C6	0.2660 (2)	0.57896 (10)	−0.11059 (18)	0.0307 (5)	
H6A	0.3388	0.5581	−0.1155	0.046*	
H6B	0.2498	0.6040	−0.1929	0.046*	
H6C	0.2104	0.5438	−0.0993	0.046*	
C7	0.47622 (18)	0.64859 (10)	0.03299 (19)	0.0267 (5)	
H7A	0.5229	0.6482	0.1140	0.040*	
H7B	0.4980	0.6861	−0.0243	0.040*	
H7C	0.4849	0.6059	−0.0148	0.040*	
C8	0.3886 (2)	0.75391 (10)	0.2411 (2)	0.0310 (5)	
H8A	0.3939	0.7959	0.1903	0.047*	
H8B	0.4624	0.7351	0.2563	0.047*	
H8C	0.3551	0.7631	0.3269	0.047*	
C9	0.1279 (2)	0.75006 (11)	0.2308 (2)	0.0346 (5)	
H9A	0.0632	0.7252	0.2602	0.052*	
H9B	0.1045	0.7866	0.1709	0.052*	
H9C	0.1672	0.7690	0.3085	0.052*	
C10	0.05177 (19)	0.64766 (12)	0.0111 (2)	0.0343 (5)	
H10A	0.0288	0.6877	−0.0397	0.051*	
H10B	0.0040	0.6420	0.0875	0.051*	
H10C	0.0462	0.6078	−0.0463	0.051*	
C11	0.26346 (16)	0.52506 (9)	0.43500 (15)	0.0159 (4)	
C12	0.18257 (18)	0.57674 (9)	0.44326 (17)	0.0201 (4)	
H12	0.1054	0.5698	0.4466	0.024*	
C13	0.2357 (2)	0.63998 (9)	0.44565 (17)	0.0261 (5)	
H13	0.2007	0.6826	0.4513	0.031*	
C14	0.34920 (19)	0.62892 (10)	0.43818 (18)	0.0267 (5)	
H14	0.4046	0.6627	0.4378	0.032*	
C15	0.36642 (17)	0.55903 (10)	0.43137 (17)	0.0208 (4)	
H15	0.4361	0.5377	0.4253	0.025*	
C16	0.24577 (16)	0.45016 (9)	0.41903 (16)	0.0157 (4)	
C17	0.34159 (16)	0.40901 (10)	0.48341 (16)	0.0194 (4)	
H17	0.4124	0.4229	0.4430	0.023*	
C18	0.32207 (19)	0.33371 (10)	0.45734 (19)	0.0265 (5)	
H18A	0.3184	0.3253	0.3602	0.032*	
H18B	0.3841	0.3073	0.4959	0.032*	
C19	0.2146 (2)	0.31122 (10)	0.5197 (2)	0.0297 (5)	
H19	0.2022	0.2622	0.5014	0.036*	
C20	0.11873 (19)	0.35226 (10)	0.4600 (2)	0.0277 (5)	
H20A	0.1123	0.3439	0.3629	0.033*	
H20B	0.0491	0.3381	0.5008	0.033*	
C21	0.13831 (17)	0.42731 (9)	0.48584 (17)	0.0205 (4)	
H21	0.0750	0.4537	0.4474	0.025*	
C22	0.34877 (18)	0.42069 (10)	0.63524 (17)	0.0253 (5)	
H22A	0.4112	0.3948	0.6743	0.030*	
H22B	0.3615	0.4691	0.6539	0.030*	
C23	0.2225 (2)	0.32297 (11)	0.6709 (2)	0.0338 (5)	
H23A	0.1536	0.3082	0.7126	0.041*	
H23B	0.2840	0.2963	0.7101	0.041*	
C24	0.14558 (18)	0.43886 (11)	0.63770 (18)	0.0267 (5)	
H24A	0.1568	0.4874	0.6566	0.032*	
H24B	0.0760	0.4247	0.6784	0.032*	
C25	0.24165 (19)	0.39807 (11)	0.69803 (18)	0.0286 (5)	
H25	0.2464	0.4059	0.7964	0.034*	
C26	0.11305 (18)	0.51058 (10)	0.1682 (2)	0.0223 (4)	
H26A	0.091 (2)	0.5577 (12)	0.195 (2)	0.033*	
H26B	0.100 (2)	0.5063 (11)	0.073 (2)	0.033*	
H26C	0.060 (2)	0.4816 (12)	0.212 (2)	0.033*	
C27	0.38455 (18)	0.49812 (10)	0.1606 (2)	0.0218 (4)	
H27A	0.4231 (19)	0.5418 (12)	0.172 (2)	0.033*	
H27B	0.3890 (19)	0.4872 (11)	0.066 (2)	0.033*	
H27C	0.433 (2)	0.4675 (12)	0.206 (2)	0.033*	
C28	0.2331 (2)	0.36873 (10)	0.10736 (18)	0.0268 (5)	
H28A	0.1724	0.3407	0.1380	0.040*	
H28B	0.3022	0.3435	0.1157	0.040*	
H28C	0.2196	0.3811	0.0139	0.040*	

### (1) [µ-1(η5)-(Adamantan-1-yl-2κC1)cycylopentadienyl]di-µ2-methyl-methyl-2κC-[1(η5)-pentamethylcyclopentadienyl]-aluminiumtitanium(III) Atomic displacement parameters (Å^2^)

**Table:**

	*U*^11^	*U*^22^	*U*^33^	*U*^12^	*U*^13^	*U*^23^
Ti1	0.0176 (2)	0.01180 (14)	0.01316 (15)	−0.00042 (14)	−0.00005 (11)	−0.00074 (12)
Al1	0.0228 (4)	0.0132 (2)	0.0145 (2)	−0.0004 (2)	−0.0004 (2)	−0.0011 (2)
C1	0.0285 (13)	0.0163 (9)	0.0133 (8)	−0.0009 (8)	−0.0002 (7)	0.0028 (7)
C2	0.0200 (12)	0.0147 (9)	0.0177 (9)	0.0001 (7)	0.0013 (7)	0.0045 (7)
C3	0.0279 (13)	0.0111 (8)	0.0180 (8)	0.0006 (8)	0.0004 (7)	0.0022 (7)
C4	0.0272 (13)	0.0150 (9)	0.0203 (9)	0.0032 (8)	0.0042 (8)	0.0048 (7)
C5	0.0214 (13)	0.0234 (10)	0.0226 (9)	−0.0009 (8)	−0.0024 (8)	0.0099 (8)
C6	0.0501 (16)	0.0239 (11)	0.0180 (9)	−0.0048 (9)	−0.0025 (9)	−0.0018 (7)
C7	0.0246 (13)	0.0269 (10)	0.0289 (10)	0.0031 (9)	0.0052 (8)	0.0084 (8)
C8	0.0463 (16)	0.0196 (10)	0.0271 (11)	−0.0093 (10)	−0.0032 (9)	0.0005 (8)
C9	0.0413 (16)	0.0280 (12)	0.0352 (12)	0.0151 (10)	0.0105 (10)	0.0058 (9)
C10	0.0266 (14)	0.0356 (12)	0.0401 (12)	−0.0044 (10)	−0.0100 (9)	0.0184 (10)
C11	0.0201 (12)	0.0187 (9)	0.0089 (8)	−0.0006 (8)	−0.0007 (7)	0.0004 (7)
C12	0.0237 (12)	0.0222 (10)	0.0145 (8)	0.0032 (8)	0.0041 (7)	0.0005 (7)
C13	0.0468 (16)	0.0156 (10)	0.0158 (9)	0.0031 (9)	0.0015 (8)	−0.0042 (7)
C14	0.0397 (16)	0.0217 (10)	0.0183 (9)	−0.0113 (9)	−0.0076 (8)	−0.0006 (7)
C15	0.0232 (13)	0.0231 (10)	0.0158 (9)	−0.0044 (8)	−0.0061 (8)	0.0027 (7)
C16	0.0159 (11)	0.0142 (8)	0.0170 (8)	−0.0014 (7)	−0.0010 (7)	0.0001 (7)
C17	0.0192 (11)	0.0207 (9)	0.0182 (8)	0.0003 (8)	−0.0007 (7)	0.0037 (7)
C18	0.0325 (14)	0.0193 (10)	0.0276 (10)	0.0056 (9)	−0.0014 (9)	0.0034 (8)
C19	0.0385 (15)	0.0186 (10)	0.0321 (11)	−0.0045 (9)	−0.0011 (9)	0.0077 (8)
C20	0.0263 (14)	0.0265 (11)	0.0303 (11)	−0.0111 (9)	0.0004 (8)	0.0062 (8)
C21	0.0174 (12)	0.0229 (10)	0.0211 (9)	−0.0014 (8)	0.0010 (7)	0.0048 (7)
C22	0.0258 (13)	0.0292 (11)	0.0205 (9)	0.0000 (9)	−0.0070 (8)	0.0052 (7)
C23	0.0365 (15)	0.0327 (12)	0.0321 (11)	−0.0028 (10)	0.0006 (10)	0.0170 (9)
C24	0.0252 (13)	0.0344 (12)	0.0210 (10)	−0.0021 (9)	0.0081 (8)	0.0028 (8)
C25	0.0344 (14)	0.0350 (11)	0.0166 (9)	−0.0024 (10)	0.0006 (8)	0.0078 (8)
C26	0.0227 (13)	0.0239 (11)	0.0202 (10)	−0.0013 (8)	−0.0014 (8)	0.0035 (8)
C27	0.0259 (13)	0.0185 (9)	0.0213 (10)	0.0031 (8)	0.0051 (8)	0.0015 (7)
C28	0.0371 (14)	0.0214 (10)	0.0220 (10)	−0.0022 (9)	0.0001 (8)	−0.0044 (8)

### (1) [µ-1(η5)-(Adamantan-1-yl-2κC1)cycylopentadienyl]di-µ2-methyl-methyl-2κC-[1(η5)-pentamethylcyclopentadienyl]-aluminiumtitanium(III) Geometric parameters (Å, º)

**Table:**

Ti1—C13	2.3481 (18)	C11—C12	1.425 (3)
Ti1—C12	2.3673 (18)	C11—C16	1.509 (2)
Ti1—C14	2.3726 (18)	C12—C13	1.411 (3)
Ti1—C1	2.3884 (17)	C12—H12	0.9500
Ti1—C2	2.3923 (17)	C13—C14	1.401 (3)
Ti1—C15	2.4025 (18)	C13—H13	0.9500
Ti1—C3	2.4032 (18)	C14—C15	1.404 (3)
Ti1—C4	2.4199 (18)	C14—H14	0.9500
Ti1—C11	2.4255 (16)	C15—H15	0.9500
Ti1—C5	2.4334 (18)	C16—C17	1.551 (3)
Ti1—C27	2.507 (2)	C16—C21	1.551 (3)
Ti1—C26	2.546 (2)	C17—C18	1.534 (3)
Ti1—Al1	2.8406 (6)	C17—C22	1.542 (2)
Ti1—H26A	2.26 (2)	C17—H17	1.0000
Al1—C28	1.9687 (19)	C18—C19	1.530 (3)
Al1—C26	2.028 (2)	C18—H18A	0.9900
Al1—C16	2.0294 (17)	C18—H18B	0.9900
Al1—C27	2.047 (2)	C19—C20	1.532 (3)
C1—C5	1.426 (3)	C19—C23	1.537 (3)
C1—C2	1.434 (3)	C19—H19	1.0000
C1—C6	1.501 (3)	C20—C21	1.529 (3)
C2—C3	1.417 (3)	C20—H20A	0.9900
C2—C7	1.499 (3)	C20—H20B	0.9900
C3—C4	1.406 (3)	C21—C24	1.542 (3)
C3—C8	1.511 (3)	C21—H21	1.0000
C4—C5	1.416 (3)	C22—C25	1.529 (3)
C4—C9	1.504 (3)	C22—H22A	0.9900
C5—C10	1.497 (3)	C22—H22B	0.9900
C6—H6A	0.9800	C23—C25	1.531 (3)
C6—H6B	0.9800	C23—H23A	0.9900
C6—H6C	0.9800	C23—H23B	0.9900
C7—H7A	0.9800	C24—C25	1.533 (3)
C7—H7B	0.9800	C24—H24A	0.9900
C7—H7C	0.9800	C24—H24B	0.9900
C8—H8A	0.9800	C25—H25	1.0000
C8—H8B	0.9800	C26—H26A	1.01 (2)
C8—H8C	0.9800	C26—H26B	0.97 (2)
C9—H9A	0.9800	C26—H26C	0.98 (2)
C9—H9B	0.9800	C27—H27A	0.99 (2)
C9—H9C	0.9800	C27—H27B	0.98 (2)
C10—H10A	0.9800	C27—H27C	0.95 (2)
C10—H10B	0.9800	C28—H28A	0.9800
C10—H10C	0.9800	C28—H28B	0.9800
C11—C15	1.423 (3)	C28—H28C	0.9800
			
C13—Ti1—C12	34.82 (7)	H7A—C7—H7B	109.5
C13—Ti1—C14	34.53 (8)	C2—C7—H7C	109.5
C12—Ti1—C14	57.43 (7)	H7A—C7—H7C	109.5
C13—Ti1—C1	139.68 (7)	H7B—C7—H7C	109.5
C12—Ti1—C1	153.91 (7)	C3—C8—H8A	109.5
C14—Ti1—C1	137.51 (7)	C3—C8—H8B	109.5
C13—Ti1—C2	120.00 (7)	H8A—C8—H8B	109.5
C12—Ti1—C2	154.65 (6)	C3—C8—H8C	109.5
C14—Ti1—C2	103.59 (7)	H8A—C8—H8C	109.5
C1—Ti1—C2	34.91 (7)	H8B—C8—H8C	109.5
C13—Ti1—C15	56.98 (7)	C4—C9—H9A	109.5
C12—Ti1—C15	56.82 (7)	C4—C9—H9B	109.5
C14—Ti1—C15	34.19 (6)	H9A—C9—H9B	109.5
C1—Ti1—C15	149.06 (7)	C4—C9—H9C	109.5
C2—Ti1—C15	118.32 (7)	H9A—C9—H9C	109.5
C13—Ti1—C3	87.44 (6)	H9B—C9—H9C	109.5
C12—Ti1—C3	120.84 (6)	C5—C10—H10A	109.5
C14—Ti1—C3	82.44 (6)	C5—C10—H10B	109.5
C1—Ti1—C3	57.18 (6)	H10A—C10—H10B	109.5
C2—Ti1—C3	34.37 (6)	C5—C10—H10C	109.5
C15—Ti1—C3	111.36 (6)	H10A—C10—H10C	109.5
C13—Ti1—C4	82.94 (6)	H10B—C10—H10C	109.5
C12—Ti1—C4	105.57 (6)	C15—C11—C12	105.67 (17)
C14—Ti1—C4	97.39 (7)	C15—C11—C16	125.91 (17)
C1—Ti1—C4	57.04 (6)	C12—C11—C16	128.15 (18)
C2—Ti1—C4	57.05 (6)	C15—C11—Ti1	71.98 (10)
C15—Ti1—C4	131.56 (7)	C12—C11—Ti1	70.48 (10)
C3—Ti1—C4	33.90 (7)	C16—C11—Ti1	117.70 (10)
C13—Ti1—C11	57.78 (6)	C13—C12—C11	108.90 (19)
C12—Ti1—C11	34.56 (6)	C13—C12—Ti1	71.85 (10)
C14—Ti1—C11	57.51 (6)	C11—C12—Ti1	74.96 (10)
C1—Ti1—C11	162.06 (6)	C13—C12—H12	125.6
C2—Ti1—C11	151.86 (7)	C11—C12—H12	125.6
C15—Ti1—C11	34.28 (6)	Ti1—C12—H12	119.4
C3—Ti1—C11	139.73 (6)	C14—C13—C12	108.14 (18)
C4—Ti1—C11	139.00 (6)	C14—C13—Ti1	73.69 (11)
C13—Ti1—C5	111.64 (7)	C12—C13—Ti1	73.33 (10)
C12—Ti1—C5	120.23 (7)	C14—C13—H13	125.9
C14—Ti1—C5	131.30 (7)	C12—C13—H13	125.9
C1—Ti1—C5	34.39 (7)	Ti1—C13—H13	118.9
C2—Ti1—C5	57.22 (7)	C13—C14—C15	107.78 (18)
C15—Ti1—C5	165.47 (7)	C13—C14—Ti1	71.78 (11)
C3—Ti1—C5	56.43 (7)	C15—C14—Ti1	74.07 (11)
C4—Ti1—C5	33.92 (7)	C13—C14—H14	126.1
C11—Ti1—C5	150.72 (7)	C15—C14—H14	126.1
C13—Ti1—C27	131.75 (7)	Ti1—C14—H14	119.9
C12—Ti1—C27	114.77 (6)	C14—C15—C11	109.50 (19)
C14—Ti1—C27	103.74 (8)	C14—C15—Ti1	71.74 (11)
C1—Ti1—C27	84.85 (7)	C11—C15—Ti1	73.74 (10)
C2—Ti1—C27	84.51 (6)	C14—C15—H15	125.2
C15—Ti1—C27	74.94 (7)	C11—C15—H15	125.2
C3—Ti1—C27	115.85 (7)	Ti1—C15—H15	120.9
C4—Ti1—C27	139.65 (6)	C11—C16—C17	111.73 (15)
C11—Ti1—C27	80.89 (6)	C11—C16—C21	111.17 (15)
C5—Ti1—C27	116.46 (7)	C17—C16—C21	107.36 (14)
C13—Ti1—C26	111.45 (7)	C11—C16—Al1	95.59 (10)
C12—Ti1—C26	79.78 (7)	C17—C16—Al1	114.39 (12)
C14—Ti1—C26	135.46 (7)	C21—C16—Al1	116.31 (12)
C1—Ti1—C26	86.63 (7)	C18—C17—C22	108.65 (15)
C2—Ti1—C26	120.95 (6)	C18—C17—C16	109.26 (15)
C15—Ti1—C26	112.82 (6)	C22—C17—C16	110.88 (15)
C3—Ti1—C26	135.35 (7)	C18—C17—H17	109.3
C4—Ti1—C26	106.17 (7)	C22—C17—H17	109.3
C11—Ti1—C26	80.66 (6)	C16—C17—H17	109.3
C5—Ti1—C26	78.93 (7)	C19—C18—C17	110.13 (16)
C27—Ti1—C26	81.97 (7)	C19—C18—H18A	109.6
C13—Ti1—Al1	115.65 (5)	C17—C18—H18A	109.6
C12—Ti1—Al1	82.96 (5)	C19—C18—H18B	109.6
C14—Ti1—Al1	112.78 (5)	C17—C18—H18B	109.6
C1—Ti1—Al1	102.43 (5)	H18A—C18—H18B	108.1
C2—Ti1—Al1	121.82 (5)	C18—C19—C20	109.55 (16)
C15—Ti1—Al1	79.34 (5)	C18—C19—C23	109.08 (18)
C3—Ti1—Al1	156.15 (5)	C20—C19—C23	109.54 (18)
C4—Ti1—Al1	147.83 (5)	C18—C19—H19	109.6
C11—Ti1—Al1	59.66 (4)	C20—C19—H19	109.6
C5—Ti1—Al1	115.01 (5)	C23—C19—H19	109.6
C27—Ti1—Al1	44.47 (5)	C21—C20—C19	109.71 (17)
C26—Ti1—Al1	43.81 (5)	C21—C20—H20A	109.7
C13—Ti1—H26A	97.0 (6)	C19—C20—H20A	109.7
C12—Ti1—H26A	72.5 (5)	C21—C20—H20B	109.7
C14—Ti1—H26A	129.0 (5)	C19—C20—H20B	109.7
C1—Ti1—H26A	86.3 (5)	H20A—C20—H20B	108.2
C2—Ti1—H26A	120.2 (5)	C20—C21—C24	108.50 (15)
C15—Ti1—H26A	121.2 (6)	C20—C21—C16	109.91 (15)
C3—Ti1—H26A	119.5 (6)	C24—C21—C16	110.84 (16)
C4—Ti1—H26A	86.6 (6)	C20—C21—H21	109.2
C11—Ti1—H26A	87.0 (6)	C24—C21—H21	109.2
C5—Ti1—H26A	66.3 (6)	C16—C21—H21	109.2
C27—Ti1—H26A	105.1 (6)	C25—C22—C17	109.61 (16)
C26—Ti1—H26A	23.3 (6)	C25—C22—H22A	109.7
Al1—Ti1—H26A	66.1 (6)	C17—C22—H22A	109.7
C28—Al1—C26	108.08 (9)	C25—C22—H22B	109.7
C28—Al1—C16	123.47 (8)	C17—C22—H22B	109.7
C26—Al1—C16	103.71 (8)	H22A—C22—H22B	108.2
C28—Al1—C27	105.47 (9)	C25—C23—C19	109.20 (15)
C26—Al1—C27	108.83 (9)	C25—C23—H23A	109.8
C16—Al1—C27	106.73 (8)	C19—C23—H23A	109.8
C28—Al1—Ti1	149.60 (6)	C25—C23—H23B	109.8
C26—Al1—Ti1	60.34 (6)	C19—C23—H23B	109.8
C16—Al1—Ti1	86.90 (5)	H23A—C23—H23B	108.3
C27—Al1—Ti1	59.09 (6)	C25—C24—C21	109.67 (16)
C5—C1—C2	107.81 (16)	C25—C24—H24A	109.7
C5—C1—C6	125.52 (18)	C21—C24—H24A	109.7
C2—C1—C6	126.12 (19)	C25—C24—H24B	109.7
C5—C1—Ti1	74.54 (10)	C21—C24—H24B	109.7
C2—C1—Ti1	72.70 (10)	H24A—C24—H24B	108.2
C6—C1—Ti1	125.20 (12)	C22—C25—C23	109.86 (18)
C3—C2—C1	107.09 (17)	C22—C25—C24	109.33 (16)
C3—C2—C7	125.68 (18)	C23—C25—C24	109.38 (18)
C1—C2—C7	126.77 (17)	C22—C25—H25	109.4
C3—C2—Ti1	73.24 (10)	C23—C25—H25	109.4
C1—C2—Ti1	72.40 (10)	C24—C25—H25	109.4
C7—C2—Ti1	125.83 (12)	Al1—C26—Ti1	75.85 (7)
C4—C3—C2	109.00 (16)	Al1—C26—H26A	133.6 (13)
C4—C3—C8	125.40 (18)	Ti1—C26—H26A	62.1 (14)
C2—C3—C8	125.0 (2)	Al1—C26—H26B	107.4 (14)
C4—C3—Ti1	73.70 (10)	Ti1—C26—H26B	114.7 (14)
C2—C3—Ti1	72.40 (10)	H26A—C26—H26B	107.4 (18)
C8—C3—Ti1	126.91 (12)	Al1—C26—H26C	94.0 (14)
C3—C4—C5	108.26 (16)	Ti1—C26—H26C	138.2 (13)
C3—C4—C9	126.24 (18)	H26A—C26—H26C	104.1 (18)
C5—C4—C9	125.0 (2)	H26B—C26—H26C	107.1 (19)
C3—C4—Ti1	72.40 (10)	Al1—C27—Ti1	76.44 (7)
C5—C4—Ti1	73.56 (11)	Al1—C27—H27A	140.1 (13)
C9—C4—Ti1	126.44 (13)	Ti1—C27—H27A	65.5 (14)
C4—C5—C1	107.81 (18)	Al1—C27—H27B	103.6 (14)
C4—C5—C10	124.33 (19)	Ti1—C27—H27B	120.1 (13)
C1—C5—C10	127.44 (18)	H27A—C27—H27B	105.4 (18)
C4—C5—Ti1	72.52 (10)	Al1—C27—H27C	94.9 (14)
C1—C5—Ti1	71.08 (10)	Ti1—C27—H27C	133.8 (13)
C10—C5—Ti1	127.77 (13)	H27A—C27—H27C	102.7 (19)
C1—C6—H6A	109.5	H27B—C27—H27C	106.2 (19)
C1—C6—H6B	109.5	Al1—C28—H28A	109.5
H6A—C6—H6B	109.5	Al1—C28—H28B	109.5
C1—C6—H6C	109.5	H28A—C28—H28B	109.5
H6A—C6—H6C	109.5	Al1—C28—H28C	109.5
H6B—C6—H6C	109.5	H28A—C28—H28C	109.5
C2—C7—H7A	109.5	H28B—C28—H28C	109.5
C2—C7—H7B	109.5		
			
C5—C1—C2—C3	1.29 (19)	C12—C13—C14—Ti1	65.77 (12)
C6—C1—C2—C3	173.12 (17)	C13—C14—C15—C11	−0.2 (2)
Ti1—C1—C2—C3	−65.54 (12)	Ti1—C14—C15—C11	−64.56 (12)
C5—C1—C2—C7	−171.20 (17)	C13—C14—C15—Ti1	64.34 (13)
C6—C1—C2—C7	0.6 (3)	C12—C11—C15—C14	0.43 (19)
Ti1—C1—C2—C7	121.97 (18)	C16—C11—C15—C14	174.87 (15)
C5—C1—C2—Ti1	66.83 (12)	Ti1—C11—C15—C14	63.28 (13)
C6—C1—C2—Ti1	−121.34 (18)	C12—C11—C15—Ti1	−62.86 (11)
C1—C2—C3—C4	−0.15 (19)	C16—C11—C15—Ti1	111.59 (15)
C7—C2—C3—C4	172.44 (16)	C15—C11—C16—C17	35.8 (2)
Ti1—C2—C3—C4	−65.13 (12)	C12—C11—C16—C17	−150.96 (16)
C1—C2—C3—C8	−171.86 (16)	Ti1—C11—C16—C17	122.91 (13)
C7—C2—C3—C8	0.7 (3)	C15—C11—C16—C21	155.76 (16)
Ti1—C2—C3—C8	123.17 (18)	C12—C11—C16—C21	−31.0 (2)
C1—C2—C3—Ti1	64.97 (12)	Ti1—C11—C16—C21	−117.17 (14)
C7—C2—C3—Ti1	−122.43 (17)	C15—C11—C16—Al1	−83.21 (17)
C2—C3—C4—C5	−1.1 (2)	C12—C11—C16—Al1	89.99 (17)
C8—C3—C4—C5	170.61 (17)	Ti1—C11—C16—Al1	3.86 (14)
Ti1—C3—C4—C5	−65.34 (13)	C11—C16—C17—C18	−176.93 (14)
C2—C3—C4—C9	−173.03 (17)	C21—C16—C17—C18	60.94 (18)
C8—C3—C4—C9	−1.4 (3)	Al1—C16—C17—C18	−69.73 (17)
Ti1—C3—C4—C9	122.69 (18)	C11—C16—C17—C22	63.35 (19)
C2—C3—C4—Ti1	64.29 (12)	C21—C16—C17—C22	−58.78 (19)
C8—C3—C4—Ti1	−124.05 (18)	Al1—C16—C17—C22	170.55 (13)
C3—C4—C5—C1	1.8 (2)	C22—C17—C18—C19	60.1 (2)
C9—C4—C5—C1	173.95 (17)	C16—C17—C18—C19	−60.95 (19)
Ti1—C4—C5—C1	−62.73 (12)	C17—C18—C19—C20	59.2 (2)
C3—C4—C5—C10	−171.19 (17)	C17—C18—C19—C23	−60.7 (2)
C9—C4—C5—C10	0.9 (3)	C18—C19—C20—C21	−58.9 (2)
Ti1—C4—C5—C10	124.23 (18)	C23—C19—C20—C21	60.7 (2)
C3—C4—C5—Ti1	64.57 (12)	C19—C20—C21—C24	−60.5 (2)
C9—C4—C5—Ti1	−123.32 (18)	C19—C20—C21—C16	60.8 (2)
C2—C1—C5—C4	−1.9 (2)	C11—C16—C21—C20	176.23 (15)
C6—C1—C5—C4	−173.83 (17)	C17—C16—C21—C20	−61.29 (19)
Ti1—C1—C5—C4	63.66 (12)	Al1—C16—C21—C20	68.30 (18)
C2—C1—C5—C10	170.82 (17)	C11—C16—C21—C24	−63.84 (19)
C6—C1—C5—C10	−1.1 (3)	C17—C16—C21—C24	58.64 (19)
Ti1—C1—C5—C10	−123.58 (19)	Al1—C16—C21—C24	−171.78 (13)
C2—C1—C5—Ti1	−65.60 (12)	C18—C17—C22—C25	−59.5 (2)
C6—C1—C5—Ti1	122.50 (18)	C16—C17—C22—C25	60.6 (2)
C15—C11—C12—C13	−0.48 (18)	C18—C19—C23—C25	60.0 (2)
C16—C11—C12—C13	−174.75 (15)	C20—C19—C23—C25	−59.9 (2)
Ti1—C11—C12—C13	−64.34 (12)	C20—C21—C24—C25	60.5 (2)
C15—C11—C12—Ti1	63.87 (11)	C16—C21—C24—C25	−60.3 (2)
C16—C11—C12—Ti1	−110.41 (16)	C17—C22—C25—C23	60.2 (2)
C11—C12—C13—C14	0.4 (2)	C17—C22—C25—C24	−59.9 (2)
Ti1—C12—C13—C14	−66.01 (13)	C19—C23—C25—C22	−60.2 (2)
C11—C12—C13—Ti1	66.37 (12)	C19—C23—C25—C24	59.8 (2)
C12—C13—C14—C15	−0.1 (2)	C21—C24—C25—C22	59.8 (2)
Ti1—C13—C14—C15	−65.86 (13)	C21—C24—C25—C23	−60.5 (2)

### (2) [µ-1(η5)-(Adamantan-1-yl-2κC1)cycylopentadienyl]di-µ2-methyl-methyl-2κC-[1(η5)-pentamethylcyclopentadienyl]-galliumtitanium(III) Crystal data

**Table:**

[GaTi(CH_3_)_3_(C_10_H_15_)(C_15_H_18_)]	*F*(000) = 1052
*M**_r_* = 496.23	*D*_x_ = 1.358 Mg m^−^^3^
Monoclinic, *P*2_1_/*c*	Mo *K*α radiation, λ = 0.71073 Å
*a* = 12.1445 (8) Å	Cell parameters from 8000 reflections
*b* = 19.9196 (7) Å	θ = 2.6–28.4°
*c* = 10.0350 (4) Å	µ = 1.45 mm^−^^1^
β = 91.400 (7)°	*T* = 153 K
*V* = 2426.9 (2) Å^3^	Block, green
*Z* = 4	0.50 × 0.30 × 0.29 mm

### (2) [µ-1(η5)-(Adamantan-1-yl-2κC1)cycylopentadienyl]di-µ2-methyl-methyl-2κC-[1(η5)-pentamethylcyclopentadienyl]-galliumtitanium(III) Data collection

**Table:**

Stoe IPDS diffractometer	4830 reflections with *I* > 2σ(*I*)
Radiation source: sealed tube	*R*_int_ = 0.042
ω–scans	θ_max_ = 28.3°, θ_min_ = 2.7°
Absorption correction: numerical (*X-RED*; Stoe, 1999)	*h* = −16→16
*T*_min_ = 0.571, *T*_max_ = 0.717	*k* = −26→26
28356 measured reflections	*l* = −13→13
5895 independent reflections	

### (2) [µ-1(η5)-(Adamantan-1-yl-2κC1)cycylopentadienyl]di-µ2-methyl-methyl-2κC-[1(η5)-pentamethylcyclopentadienyl]-galliumtitanium(III) Refinement

**Table:**

Refinement on *F*^2^	0 restraints
Least-squares matrix: full	Hydrogen site location: mixed
*R*[*F*^2^ > 2σ(*F*^2^)] = 0.026	H atoms treated by a mixture of independent and constrained refinement
*wR*(*F*^2^) = 0.065	*w* = 1/[σ^2^(*F*_o_^2^) + (0.045*P*)^2^] where *P* = (*F*_o_^2^ + 2*F*_c_^2^)/3
*S* = 0.94	(Δ/σ)_max_ = 0.001
5895 reflections	Δρ_max_ = 0.57 e Å^−^^3^
295 parameters	Δρ_min_ = −0.32 e Å^−^^3^

### (2) [µ-1(η5)-(Adamantan-1-yl-2κC1)cycylopentadienyl]di-µ2-methyl-methyl-2κC-[1(η5)-pentamethylcyclopentadienyl]-galliumtitanium(III) Special details

**Table:**

Geometry. All esds (except the esd in the dihedral angle between two l.s. planes) are estimated using the full covariance matrix. The cell esds are taken into account individually in the estimation of esds in distances, angles and torsion angles; correlations between esds in cell parameters are only used when they are defined by crystal symmetry. An approximate (isotropic) treatment of cell esds is used for estimating esds involving l.s. planes.

### (2) [µ-1(η5)-(Adamantan-1-yl-2κC1)cycylopentadienyl]di-µ2-methyl-methyl-2κC-[1(η5)-pentamethylcyclopentadienyl]-galliumtitanium(III) Fractional atomic coordinates and isotropic or equivalent isotropic displacement parameters (Å^2^)

**Table:**

	*x*	*y*	*z*	*U*_iso_*/*U*_eq_	
Ti1	0.26510 (2)	0.59328 (2)	0.23594 (3)	0.01499 (6)	
Ga1	0.24185 (2)	0.44945 (2)	0.21612 (2)	0.01868 (5)	
C1	0.26371 (12)	0.62639 (7)	0.00683 (16)	0.0200 (3)	
C2	0.35740 (11)	0.65699 (7)	0.07047 (15)	0.0187 (3)	
C3	0.31964 (12)	0.70397 (7)	0.16453 (16)	0.0201 (3)	
C4	0.20233 (12)	0.70289 (7)	0.16042 (16)	0.0218 (3)	
C5	0.16791 (12)	0.65626 (8)	0.06199 (16)	0.0220 (3)	
C6	0.26467 (16)	0.57887 (8)	−0.10972 (18)	0.0306 (4)	
H6A	0.3388	0.5603	−0.1187	0.046*	
H6B	0.2434	0.6031	−0.1914	0.046*	
H6C	0.2124	0.5423	−0.0950	0.046*	
C7	0.47592 (12)	0.64853 (8)	0.03272 (18)	0.0263 (3)	
H7A	0.5226	0.6463	0.1137	0.039*	
H7B	0.4984	0.6868	−0.0215	0.039*	
H7C	0.4839	0.6070	−0.0185	0.039*	
C8	0.38924 (15)	0.75375 (8)	0.24158 (19)	0.0305 (4)	
H8A	0.3962	0.7951	0.1894	0.046*	
H8B	0.4625	0.7346	0.2593	0.046*	
H8C	0.3544	0.7640	0.3262	0.046*	
C9	0.12758 (15)	0.75012 (9)	0.2327 (2)	0.0341 (4)	
H9A	0.0645	0.7251	0.2662	0.051*	
H9B	0.1014	0.7852	0.1713	0.051*	
H9C	0.1682	0.7707	0.3077	0.051*	
C10	0.05046 (13)	0.64805 (9)	0.0134 (2)	0.0336 (4)	
H10A	0.0258	0.6894	−0.0312	0.050*	
H10B	0.0035	0.6390	0.0894	0.050*	
H10C	0.0456	0.6105	−0.0495	0.050*	
C11	0.26365 (12)	0.52545 (7)	0.43654 (15)	0.0169 (3)	
C12	0.18259 (13)	0.57730 (8)	0.44522 (16)	0.0220 (3)	
H12	0.1053	0.5705	0.4491	0.026*	
C13	0.23655 (15)	0.64068 (8)	0.44711 (17)	0.0276 (3)	
H13	0.2018	0.6833	0.4528	0.033*	
C14	0.35007 (15)	0.62917 (8)	0.43912 (17)	0.0275 (3)	
H14	0.4059	0.6626	0.4382	0.033*	
C15	0.36680 (12)	0.55911 (7)	0.43266 (16)	0.0211 (3)	
H15	0.4364	0.5377	0.4266	0.025*	
C16	0.24577 (11)	0.45104 (7)	0.41968 (15)	0.0160 (3)	
C17	0.34130 (11)	0.40929 (7)	0.48259 (16)	0.0199 (3)	
H17	0.4123	0.4234	0.4427	0.024*	
C18	0.32215 (14)	0.33399 (8)	0.45491 (19)	0.0268 (3)	
H18A	0.3188	0.3260	0.3575	0.032*	
H18B	0.3842	0.3076	0.4934	0.032*	
C19	0.21393 (15)	0.31129 (8)	0.51680 (19)	0.0307 (4)	
H19	0.2013	0.2626	0.4975	0.037*	
C20	0.11816 (13)	0.35279 (8)	0.45715 (19)	0.0279 (3)	
H20A	0.0482	0.3385	0.4971	0.033*	
H20B	0.1122	0.3451	0.3598	0.033*	
C21	0.13800 (12)	0.42791 (8)	0.48504 (16)	0.0211 (3)	
H21	0.0748	0.4545	0.4470	0.025*	
C22	0.34832 (13)	0.41990 (8)	0.63446 (17)	0.0256 (3)	
H22A	0.4107	0.3938	0.6729	0.031*	
H22B	0.3615	0.4680	0.6543	0.031*	
C23	0.22157 (16)	0.32224 (9)	0.6678 (2)	0.0360 (4)	
H23A	0.2831	0.2954	0.7065	0.043*	
H23B	0.1525	0.3073	0.7089	0.043*	
C24	0.14504 (14)	0.43846 (9)	0.63692 (18)	0.0283 (3)	
H24A	0.1565	0.4867	0.6569	0.034*	
H24B	0.0751	0.4243	0.6771	0.034*	
C25	0.24086 (14)	0.39729 (9)	0.69729 (18)	0.0300 (4)	
H25	0.2455	0.4046	0.7959	0.036*	
C26	0.10953 (13)	0.50957 (8)	0.16647 (18)	0.0233 (3)	
H26A	0.0920 (18)	0.5522 (11)	0.194 (2)	0.035*	
H26B	0.0986 (18)	0.5057 (11)	0.077 (3)	0.035*	
H26C	0.0531 (19)	0.4848 (11)	0.206 (2)	0.035*	
C27	0.38617 (12)	0.49823 (8)	0.15818 (17)	0.0185 (3)	
H27A	0.4057 (18)	0.5323 (11)	0.169 (2)	0.028*	
H27B	0.3895 (16)	0.4900 (10)	0.076 (2)	0.028*	
H27C	0.4357 (17)	0.4692 (10)	0.206 (2)	0.028*	
C28	0.23418 (15)	0.36701 (8)	0.10482 (19)	0.0298 (4)	
H28A	0.1762	0.3374	0.1374	0.045*	
H28B	0.3051	0.3436	0.1101	0.045*	
H28C	0.2174	0.3793	0.0120	0.045*	

### (2) [µ-1(η5)-(Adamantan-1-yl-2κC1)cycylopentadienyl]di-µ2-methyl-methyl-2κC-[1(η5)-pentamethylcyclopentadienyl]-galliumtitanium(III) Atomic displacement parameters (Å^2^)

**Table:**

	*U*^11^	*U*^22^	*U*^33^	*U*^12^	*U*^13^	*U*^23^
Ti1	0.01601 (11)	0.01426 (11)	0.01467 (14)	−0.00026 (8)	−0.00024 (9)	−0.00099 (9)
Ga1	0.02268 (8)	0.01700 (8)	0.01628 (9)	0.00069 (6)	−0.00110 (6)	−0.00145 (6)
C1	0.0237 (7)	0.0198 (7)	0.0162 (8)	−0.0006 (5)	−0.0012 (6)	0.0024 (5)
C2	0.0195 (6)	0.0185 (6)	0.0181 (8)	0.0002 (5)	0.0019 (5)	0.0040 (5)
C3	0.0240 (7)	0.0166 (6)	0.0196 (8)	−0.0012 (5)	0.0001 (6)	0.0022 (5)
C4	0.0236 (7)	0.0189 (7)	0.0230 (8)	0.0044 (5)	0.0040 (6)	0.0051 (6)
C5	0.0198 (7)	0.0234 (7)	0.0228 (9)	−0.0001 (5)	−0.0017 (6)	0.0079 (6)
C6	0.0467 (10)	0.0263 (8)	0.0186 (9)	−0.0027 (7)	−0.0031 (7)	−0.0022 (6)
C7	0.0210 (7)	0.0296 (8)	0.0285 (9)	0.0032 (6)	0.0057 (6)	0.0071 (6)
C8	0.0410 (9)	0.0209 (7)	0.0293 (10)	−0.0085 (7)	−0.0037 (7)	−0.0007 (6)
C9	0.0379 (9)	0.0302 (8)	0.0347 (11)	0.0139 (7)	0.0106 (8)	0.0035 (7)
C10	0.0214 (7)	0.0389 (9)	0.0400 (11)	−0.0047 (6)	−0.0089 (7)	0.0157 (8)
C11	0.0198 (6)	0.0186 (6)	0.0124 (7)	−0.0002 (5)	−0.0010 (5)	−0.0001 (5)
C12	0.0253 (7)	0.0241 (7)	0.0168 (8)	0.0039 (6)	0.0035 (6)	−0.0015 (6)
C13	0.0475 (10)	0.0183 (7)	0.0170 (8)	0.0036 (6)	0.0006 (7)	−0.0036 (6)
C14	0.0392 (9)	0.0219 (7)	0.0210 (9)	−0.0097 (6)	−0.0083 (7)	−0.0007 (6)
C15	0.0225 (7)	0.0226 (7)	0.0180 (8)	−0.0048 (5)	−0.0057 (6)	0.0017 (5)
C16	0.0150 (6)	0.0165 (6)	0.0167 (7)	−0.0006 (5)	−0.0007 (5)	0.0012 (5)
C17	0.0173 (6)	0.0213 (7)	0.0210 (8)	0.0012 (5)	−0.0020 (5)	0.0041 (6)
C18	0.0313 (8)	0.0198 (7)	0.0291 (10)	0.0052 (6)	−0.0024 (7)	0.0051 (6)
C19	0.0353 (8)	0.0215 (7)	0.0351 (10)	−0.0060 (6)	−0.0029 (7)	0.0089 (7)
C20	0.0255 (7)	0.0250 (8)	0.0330 (10)	−0.0094 (6)	−0.0007 (6)	0.0059 (7)
C21	0.0161 (6)	0.0243 (7)	0.0230 (9)	−0.0027 (5)	0.0006 (6)	0.0031 (6)
C22	0.0243 (7)	0.0304 (8)	0.0217 (9)	−0.0011 (6)	−0.0056 (6)	0.0044 (6)
C23	0.0385 (9)	0.0350 (9)	0.0345 (11)	−0.0036 (7)	−0.0007 (8)	0.0180 (8)
C24	0.0265 (8)	0.0362 (9)	0.0226 (9)	−0.0013 (6)	0.0078 (6)	0.0037 (7)
C25	0.0336 (8)	0.0389 (9)	0.0176 (9)	−0.0036 (7)	0.0009 (7)	0.0086 (7)
C26	0.0210 (7)	0.0271 (8)	0.0216 (9)	0.0014 (6)	−0.0019 (6)	0.0035 (6)
C27	0.0180 (6)	0.0173 (7)	0.0205 (8)	−0.0001 (5)	0.0062 (6)	0.0016 (6)
C28	0.0369 (9)	0.0261 (8)	0.0264 (10)	−0.0022 (6)	0.0004 (7)	−0.0091 (7)

### (2) [µ-1(η5)-(Adamantan-1-yl-2κC1)cycylopentadienyl]di-µ2-methyl-methyl-2κC-[1(η5)-pentamethylcyclopentadienyl]-galliumtitanium(III) Geometric parameters (Å, º)

**Table:**

Ti1—C13	2.3533 (17)	C11—C12	1.431 (2)
Ti1—C12	2.3704 (16)	C11—C16	1.5069 (19)
Ti1—C14	2.3728 (16)	C12—C13	1.422 (2)
Ti1—C2	2.3906 (15)	C12—H12	0.9500
Ti1—C1	2.3916 (16)	C13—C14	1.402 (3)
Ti1—C15	2.4013 (15)	C13—H13	0.9500
Ti1—C3	2.4160 (14)	C14—C15	1.412 (2)
Ti1—C11	2.4248 (15)	C14—H14	0.9500
Ti1—C4	2.4279 (14)	C15—H15	0.9500
Ti1—C5	2.4318 (15)	C16—C21	1.5483 (19)
Ti1—C27	2.5322 (16)	C16—C17	1.5493 (19)
Ti1—C26	2.6023 (17)	C17—C22	1.539 (2)
Ti1—Ga1	2.8852 (3)	C17—C18	1.542 (2)
Ti1—H27A	2.22 (2)	C17—H17	1.0000
Ga1—C28	1.9869 (16)	C18—C19	1.535 (2)
Ga1—C16	2.0423 (15)	C18—H18A	0.9900
Ga1—C26	2.0556 (15)	C18—H18B	0.9900
Ga1—C27	2.0985 (15)	C19—C23	1.531 (3)
C1—C2	1.428 (2)	C19—C20	1.536 (2)
C1—C5	1.430 (2)	C19—H19	1.0000
C1—C6	1.505 (2)	C20—C21	1.540 (2)
C2—C3	1.414 (2)	C20—H20A	0.9900
C2—C7	1.507 (2)	C20—H20B	0.9900
C3—C4	1.424 (2)	C21—C24	1.539 (2)
C3—C8	1.504 (2)	C21—H21	1.0000
C4—C5	1.412 (2)	C22—C25	1.531 (2)
C4—C9	1.506 (2)	C22—H22A	0.9900
C5—C10	1.505 (2)	C22—H22B	0.9900
C6—H6A	0.9800	C23—C25	1.541 (3)
C6—H6B	0.9800	C23—H23A	0.9900
C6—H6C	0.9800	C23—H23B	0.9900
C7—H7A	0.9800	C24—C25	1.536 (2)
C7—H7B	0.9800	C24—H24A	0.9900
C7—H7C	0.9800	C24—H24B	0.9900
C8—H8A	0.9800	C25—H25	1.0000
C8—H8B	0.9800	C26—H26A	0.92 (2)
C8—H8C	0.9800	C26—H26B	0.90 (2)
C9—H9A	0.9800	C26—H26C	0.94 (2)
C9—H9B	0.9800	C27—H27A	0.73 (2)
C9—H9C	0.9800	C27—H27B	0.84 (2)
C10—H10A	0.9800	C27—H27C	0.96 (2)
C10—H10B	0.9800	C28—H28A	0.9800
C10—H10C	0.9800	C28—H28B	0.9800
C11—C15	1.422 (2)	C28—H28C	0.9800
			
C13—Ti1—C12	35.05 (6)	H7A—C7—H7B	109.5
C13—Ti1—C14	34.51 (6)	C2—C7—H7C	109.5
C12—Ti1—C14	57.56 (6)	H7A—C7—H7C	109.5
C13—Ti1—C2	119.63 (6)	H7B—C7—H7C	109.5
C12—Ti1—C2	154.45 (5)	C3—C8—H8A	109.5
C14—Ti1—C2	103.62 (6)	C3—C8—H8B	109.5
C13—Ti1—C1	139.23 (5)	H8A—C8—H8B	109.5
C12—Ti1—C1	153.58 (5)	C3—C8—H8C	109.5
C14—Ti1—C1	137.36 (5)	H8A—C8—H8C	109.5
C2—Ti1—C1	34.74 (5)	H8B—C8—H8C	109.5
C13—Ti1—C15	57.16 (6)	C4—C9—H9A	109.5
C12—Ti1—C15	56.89 (5)	C4—C9—H9B	109.5
C14—Ti1—C15	34.40 (5)	H9A—C9—H9B	109.5
C2—Ti1—C15	118.75 (5)	C4—C9—H9C	109.5
C1—Ti1—C15	149.30 (5)	H9A—C9—H9C	109.5
C13—Ti1—C3	87.04 (6)	H9B—C9—H9C	109.5
C12—Ti1—C3	120.68 (5)	C5—C10—H10A	109.5
C14—Ti1—C3	82.14 (5)	C5—C10—H10B	109.5
C2—Ti1—C3	34.20 (5)	H10A—C10—H10B	109.5
C1—Ti1—C3	57.23 (5)	C5—C10—H10C	109.5
C15—Ti1—C3	111.30 (5)	H10A—C10—H10C	109.5
C13—Ti1—C11	58.12 (5)	H10B—C10—H10C	109.5
C12—Ti1—C11	34.70 (5)	C15—C11—C12	105.61 (13)
C14—Ti1—C11	57.75 (5)	C15—C11—C16	125.82 (13)
C2—Ti1—C11	152.31 (5)	C12—C11—C16	128.26 (13)
C1—Ti1—C11	162.12 (5)	C15—C11—Ti1	71.96 (9)
C15—Ti1—C11	34.28 (5)	C12—C11—Ti1	70.57 (9)
C3—Ti1—C11	139.68 (5)	C16—C11—Ti1	117.31 (10)
C13—Ti1—C4	82.43 (6)	C13—C12—C11	108.91 (14)
C12—Ti1—C4	105.13 (5)	C13—C12—Ti1	71.82 (9)
C14—Ti1—C4	97.12 (6)	C11—C12—Ti1	74.73 (9)
C2—Ti1—C4	56.96 (5)	C13—C12—H12	125.5
C1—Ti1—C4	57.12 (5)	C11—C12—H12	125.5
C15—Ti1—C4	131.51 (5)	Ti1—C12—H12	119.7
C3—Ti1—C4	34.20 (5)	C14—C13—C12	107.91 (14)
C11—Ti1—C4	138.74 (5)	C14—C13—Ti1	73.51 (10)
C13—Ti1—C5	111.09 (6)	C12—C13—Ti1	73.13 (9)
C12—Ti1—C5	119.80 (5)	C14—C13—H13	126.0
C14—Ti1—C5	130.89 (6)	C12—C13—H13	126.0
C2—Ti1—C5	56.98 (5)	Ti1—C13—H13	119.2
C1—Ti1—C5	34.49 (5)	C13—C14—C15	107.89 (14)
C15—Ti1—C5	165.29 (5)	C13—C14—Ti1	71.99 (9)
C3—Ti1—C5	56.50 (5)	C15—C14—Ti1	73.91 (9)
C11—Ti1—C5	150.48 (5)	C13—C14—H14	126.1
C4—Ti1—C5	33.78 (5)	C15—C14—H14	126.1
C13—Ti1—C27	132.61 (6)	Ti1—C14—H14	119.9
C12—Ti1—C27	115.71 (5)	C14—C15—C11	109.69 (14)
C14—Ti1—C27	104.27 (6)	C14—C15—Ti1	71.69 (9)
C2—Ti1—C27	84.10 (5)	C11—C15—Ti1	73.77 (8)
C1—Ti1—C27	84.29 (5)	C14—C15—H15	125.2
C15—Ti1—C27	75.55 (6)	C11—C15—H15	125.2
C3—Ti1—C27	115.18 (5)	Ti1—C15—H15	121.0
C11—Ti1—C27	81.67 (5)	C11—C16—C21	111.47 (12)
C4—Ti1—C27	139.16 (5)	C11—C16—C17	112.20 (11)
C5—Ti1—C27	116.09 (6)	C21—C16—C17	107.46 (12)
C13—Ti1—C26	112.11 (6)	C11—C16—Ga1	97.31 (9)
C12—Ti1—C26	80.29 (6)	C21—C16—Ga1	114.82 (10)
C14—Ti1—C26	136.13 (6)	C17—C16—Ga1	113.46 (10)
C2—Ti1—C26	120.25 (5)	C22—C17—C18	108.45 (13)
C1—Ti1—C26	86.05 (5)	C22—C17—C16	110.65 (12)
C15—Ti1—C26	113.21 (5)	C18—C17—C16	109.91 (12)
C3—Ti1—C26	135.06 (5)	C22—C17—H17	109.3
C11—Ti1—C26	81.10 (5)	C18—C17—H17	109.3
C4—Ti1—C26	105.80 (5)	C16—C17—H17	109.3
C5—Ti1—C26	78.57 (5)	C19—C18—C17	109.91 (14)
C27—Ti1—C26	82.04 (5)	C19—C18—H18A	109.7
C13—Ti1—Ga1	116.37 (4)	C17—C18—H18A	109.7
C12—Ti1—Ga1	83.39 (4)	C19—C18—H18B	109.7
C14—Ti1—Ga1	113.47 (4)	C17—C18—H18B	109.7
C2—Ti1—Ga1	121.73 (4)	H18A—C18—H18B	108.2
C1—Ti1—Ga1	102.07 (4)	C23—C19—C18	109.06 (15)
C15—Ti1—Ga1	79.79 (4)	C23—C19—C20	109.62 (16)
C3—Ti1—Ga1	155.90 (4)	C18—C19—C20	109.35 (13)
C11—Ti1—Ga1	60.08 (3)	C23—C19—H19	109.6
C4—Ti1—Ga1	147.35 (4)	C18—C19—H19	109.6
C5—Ti1—Ga1	114.70 (4)	C20—C19—H19	109.6
C27—Ti1—Ga1	45.00 (3)	C19—C20—C21	109.78 (13)
C26—Ti1—Ga1	43.62 (3)	C19—C20—H20A	109.7
C13—Ti1—H27A	128.8 (6)	C21—C20—H20A	109.7
C12—Ti1—H27A	122.9 (6)	C19—C20—H20B	109.7
C14—Ti1—H27A	96.0 (6)	C21—C20—H20B	109.7
C2—Ti1—H27A	72.6 (6)	H20A—C20—H20B	108.2
C1—Ti1—H27A	81.1 (6)	C24—C21—C20	108.49 (13)
C15—Ti1—H27A	73.2 (6)	C24—C21—C16	110.60 (12)
C3—Ti1—H27A	101.0 (6)	C20—C21—C16	110.01 (13)
C11—Ti1—H27A	88.2 (6)	C24—C21—H21	109.2
C4—Ti1—H27A	129.5 (6)	C20—C21—H21	109.2
C5—Ti1—H27A	115.4 (6)	C16—C21—H21	109.2
C27—Ti1—H27A	15.8 (6)	C25—C22—C17	109.89 (13)
C26—Ti1—H27A	97.3 (6)	C25—C22—H22A	109.7
Ga1—Ti1—H27A	60.6 (6)	C17—C22—H22A	109.7
C28—Ga1—C16	125.07 (7)	C25—C22—H22B	109.7
C28—Ga1—C26	108.64 (7)	C17—C22—H22B	109.7
C16—Ga1—C26	103.44 (6)	H22A—C22—H22B	108.2
C28—Ga1—C27	104.77 (7)	C19—C23—C25	109.49 (14)
C16—Ga1—C27	105.71 (6)	C19—C23—H23A	109.8
C26—Ga1—C27	108.45 (6)	C25—C23—H23A	109.8
C28—Ga1—Ti1	149.61 (6)	C19—C23—H23B	109.8
C16—Ga1—Ti1	85.17 (4)	C25—C23—H23B	109.8
C26—Ga1—Ti1	60.85 (5)	H23A—C23—H23B	108.2
C27—Ga1—Ti1	58.56 (4)	C25—C24—C21	109.93 (14)
C2—C1—C5	107.23 (13)	C25—C24—H24A	109.7
C2—C1—C6	126.51 (14)	C21—C24—H24A	109.7
C5—C1—C6	125.75 (14)	C25—C24—H24B	109.7
C2—C1—Ti1	72.59 (9)	C21—C24—H24B	109.7
C5—C1—Ti1	74.30 (9)	H24A—C24—H24B	108.2
C6—C1—Ti1	125.01 (10)	C22—C25—C24	109.01 (13)
C3—C2—C1	108.26 (13)	C22—C25—C23	109.48 (15)
C3—C2—C7	124.70 (14)	C24—C25—C23	109.39 (15)
C1—C2—C7	126.55 (14)	C22—C25—H25	109.6
C3—C2—Ti1	73.88 (9)	C24—C25—H25	109.6
C1—C2—Ti1	72.67 (8)	C23—C25—H25	109.6
C7—C2—Ti1	125.61 (10)	Ga1—C26—Ti1	75.53 (5)
C2—C3—C4	108.13 (13)	Ga1—C26—H26A	130.7 (14)
C2—C3—C8	126.31 (14)	Ti1—C26—H26A	60.0 (14)
C4—C3—C8	125.02 (15)	Ga1—C26—H26B	106.6 (14)
C2—C3—Ti1	71.92 (8)	Ti1—C26—H26B	114.1 (14)
C4—C3—Ti1	73.36 (8)	H26A—C26—H26B	110 (2)
C8—C3—Ti1	127.13 (11)	Ga1—C26—H26C	99.6 (13)
C5—C4—C3	108.00 (13)	Ti1—C26—H26C	139.3 (14)
C5—C4—C9	125.20 (14)	H26A—C26—H26C	100.7 (19)
C3—C4—C9	126.22 (15)	H26B—C26—H26C	106.1 (19)
C5—C4—Ti1	73.26 (8)	Ga1—C27—Ti1	76.45 (5)
C3—C4—Ti1	72.44 (8)	Ga1—C27—H27A	131.8 (17)
C9—C4—Ti1	126.85 (11)	Ti1—C27—H27A	56.5 (17)
C4—C5—C1	108.35 (13)	Ga1—C27—H27B	103.9 (14)
C4—C5—C10	124.06 (15)	Ti1—C27—H27B	119.3 (14)
C1—C5—C10	127.09 (16)	H27A—C27—H27B	107 (2)
C4—C5—Ti1	72.96 (9)	Ga1—C27—H27C	95.7 (12)
C1—C5—Ti1	71.22 (8)	Ti1—C27—H27C	131.1 (13)
C10—C5—Ti1	128.02 (11)	H27A—C27—H27C	107 (2)
C1—C6—H6A	109.5	H27B—C27—H27C	109.4 (18)
C1—C6—H6B	109.5	Ga1—C28—H28A	109.5
H6A—C6—H6B	109.5	Ga1—C28—H28B	109.5
C1—C6—H6C	109.5	H28A—C28—H28B	109.5
H6A—C6—H6C	109.5	Ga1—C28—H28C	109.5
H6B—C6—H6C	109.5	H28A—C28—H28C	109.5
C2—C7—H7A	109.5	H28B—C28—H28C	109.5
C2—C7—H7B	109.5		
			
C5—C1—C2—C3	0.94 (16)	C12—C13—C14—Ti1	65.56 (12)
C6—C1—C2—C3	173.10 (15)	C13—C14—C15—C11	−0.01 (19)
Ti1—C1—C2—C3	−65.84 (10)	Ti1—C14—C15—C11	−64.46 (11)
C5—C1—C2—C7	−171.35 (14)	C13—C14—C15—Ti1	64.45 (12)
C6—C1—C2—C7	0.8 (2)	C12—C11—C15—C14	0.17 (18)
Ti1—C1—C2—C7	121.87 (15)	C16—C11—C15—C14	174.25 (14)
C5—C1—C2—Ti1	66.78 (10)	Ti1—C11—C15—C14	63.15 (12)
C6—C1—C2—Ti1	−121.06 (15)	C12—C11—C15—Ti1	−62.98 (11)
C1—C2—C3—C4	0.13 (17)	C16—C11—C15—Ti1	111.10 (15)
C7—C2—C3—C4	172.59 (14)	C15—C11—C16—C21	156.47 (15)
Ti1—C2—C3—C4	−64.91 (10)	C12—C11—C16—C21	−30.8 (2)
C1—C2—C3—C8	−171.72 (15)	Ti1—C11—C16—C21	−116.80 (11)
C7—C2—C3—C8	0.7 (2)	C15—C11—C16—C17	35.9 (2)
Ti1—C2—C3—C8	123.24 (16)	C12—C11—C16—C17	−151.37 (15)
C1—C2—C3—Ti1	65.04 (10)	Ti1—C11—C16—C17	122.62 (11)
C7—C2—C3—Ti1	−122.49 (15)	C15—C11—C16—Ga1	−83.18 (15)
C2—C3—C4—C5	−1.17 (17)	C12—C11—C16—Ga1	89.56 (16)
C8—C3—C4—C5	170.82 (15)	Ti1—C11—C16—Ga1	3.55 (10)
Ti1—C3—C4—C5	−65.14 (10)	C11—C16—C17—C22	63.70 (16)
C2—C3—C4—C9	−172.80 (15)	C21—C16—C17—C22	−59.17 (15)
C8—C3—C4—C9	−0.8 (3)	Ga1—C16—C17—C22	172.79 (9)
Ti1—C3—C4—C9	123.23 (16)	C11—C16—C17—C18	−176.55 (13)
C2—C3—C4—Ti1	63.97 (10)	C21—C16—C17—C18	60.58 (16)
C8—C3—C4—Ti1	−124.05 (16)	Ga1—C16—C17—C18	−67.47 (14)
C3—C4—C5—C1	1.75 (17)	C22—C17—C18—C19	60.40 (16)
C9—C4—C5—C1	173.49 (15)	C16—C17—C18—C19	−60.68 (17)
Ti1—C4—C5—C1	−62.84 (10)	C17—C18—C19—C23	−60.83 (17)
C3—C4—C5—C10	−170.61 (14)	C17—C18—C19—C20	59.03 (19)
C9—C4—C5—C10	1.1 (2)	C23—C19—C20—C21	60.45 (18)
Ti1—C4—C5—C10	124.79 (15)	C18—C19—C20—C21	−59.06 (19)
C3—C4—C5—Ti1	64.60 (10)	C19—C20—C21—C24	−60.25 (17)
C9—C4—C5—Ti1	−123.66 (16)	C19—C20—C21—C16	60.87 (18)
C2—C1—C5—C4	−1.67 (17)	C11—C16—C21—C24	−64.24 (16)
C6—C1—C5—C4	−173.90 (15)	C17—C16—C21—C24	59.08 (16)
Ti1—C1—C5—C4	63.97 (11)	Ga1—C16—C21—C24	−173.66 (10)
C2—C1—C5—C10	170.41 (15)	C11—C16—C21—C20	175.92 (12)
C6—C1—C5—C10	−1.8 (3)	C17—C16—C21—C20	−60.76 (16)
Ti1—C1—C5—C10	−123.96 (15)	Ga1—C16—C21—C20	66.50 (14)
C2—C1—C5—Ti1	−65.64 (10)	C18—C17—C22—C25	−59.99 (16)
C6—C1—C5—Ti1	122.13 (15)	C16—C17—C22—C25	60.63 (16)
C15—C11—C12—C13	−0.26 (18)	C18—C19—C23—C25	60.07 (18)
C16—C11—C12—C13	−174.15 (15)	C20—C19—C23—C25	−59.62 (18)
Ti1—C11—C12—C13	−64.19 (12)	C20—C21—C24—C25	60.31 (16)
C15—C11—C12—Ti1	63.92 (11)	C16—C21—C24—C25	−60.43 (17)
C16—C11—C12—Ti1	−109.97 (15)	C17—C22—C25—C24	−59.51 (18)
C11—C12—C13—C14	0.26 (19)	C17—C22—C25—C23	60.12 (17)
Ti1—C12—C13—C14	−65.81 (12)	C21—C24—C25—C22	59.47 (18)
C11—C12—C13—Ti1	66.08 (11)	C21—C24—C25—C23	−60.21 (18)
C12—C13—C14—C15	−0.16 (19)	C19—C23—C25—C22	−59.94 (18)
Ti1—C13—C14—C15	−65.72 (12)	C19—C23—C25—C24	59.45 (19)
